# Trends and Predictors of Transmitted Drug Resistance (TDR) and Clusters with TDR in a Local Belgian HIV-1 Epidemic

**DOI:** 10.1371/journal.pone.0101738

**Published:** 2014-07-08

**Authors:** Andrea-Clemencia Pineda-Peña, Yoeri Schrooten, Lore Vinken, Fossie Ferreira, Guangdi Li, Nídia Sequeira Trovão, Ricardo Khouri, Inge Derdelinckx, Paul De Munter, Claudia Kücherer, Leondios G. Kostrikis, Claus Nielsen, Kirsi Littsola, Annemarie Wensing, Maja Stanojevic, Roger Paredes, Claudia Balotta, Jan Albert, Charles Boucher, Arley Gomez-Lopez, Eric Van Wijngaerden, Marc Van Ranst, Jurgen Vercauteren, Anne-Mieke Vandamme, Kristel Van Laethem

**Affiliations:** 1 Clinical and Epidemiological Virology, Rega Institute for Medical Research, Department of Microbiology and Immunology, KU Leuven, Leuven, Belgium; 2 Clinical and Molecular Infectious Diseases Group, Faculty of Sciences and Mathematics, Universidad del Rosario, Bogotá, Colombia; 3 AIDS Reference Laboratory, University Hospitals Leuven, Leuven, Belgium; 4 Clinical Infectious and Inflammatory Disorders, Department of Microbiology and Immunology, KU Leuven, Leuven, Belgium; 5 Internal Medicine, University Hospitals Leuven, Leuven, Belgium; 6 Robert Koch-Institut, Berlin, Germany; 7 University of Cyprus, Nicosia, Cyprus; 8 Statens Serum Institut, Copenhagen, Denmark; 9 National Institute of health and welfare, Helsinki, Finland; 10 Department of Virology, University Medical Center Utrecht, The Netherlands; 11 University of Belgrade, Faculty of Medicine, Belgrade, Serbia; 12 IrsiCaixa Foundation, Badalona, Spain; 13 Luigi Sacco University Hospital, Milan, Italy; 14 Department of Microbiology, Tumor and Cell Biology, Karolinska Institutet, Stockholm, Sweden; 15 Department of Clinical Microbiology, Karolinska University Hospital, Stockholm, Sweden; 16 Erasmus MC, University Medical Center, Rotterdam, The Netherlands; 17 Centro de Malária e outras Doenças Tropicais and Unidade de Microbiologia, Instituto de Higiene e Medicina Tropical, Universidade Nova de Lisboa, Lisboa, Portugal; Centro Nacional de Microbiología - Instituto de Salud Carlos III, Spain

## Abstract

We aimed to study epidemic trends and predictors for transmitted drug resistance (TDR) in our region, its clinical impact and its association with transmission clusters. We included 778 patients from the AIDS Reference Center in Leuven (Belgium) diagnosed from 1998 to 2012. Resistance testing was performed using population-based sequencing and TDR was estimated using the WHO-2009 surveillance list. Phylogenetic analysis was performed using maximum likelihood and Bayesian techniques. The cohort was predominantly Belgian (58.4%), men who have sex with men (MSM) (42.8%), and chronically infected (86.5%). The overall TDR prevalence was 9.6% (95% confidence interval (CI): 7.7–11.9), 6.5% (CI: 5.0–8.5) for nucleoside reverse transcriptase inhibitors (NRTI), 2.2% (CI: 1.4–3.5) for non-NRTI (NNRTI), and 2.2% (CI: 1.4–3.5) for protease inhibitors. A significant parabolic trend of NNRTI-TDR was found (p = 0.019). Factors significantly associated with TDR in univariate analysis were male gender, Belgian origin, MSM, recent infection, transmission clusters and subtype B, while multivariate and Bayesian network analysis singled out subtype B as the most predictive factor of TDR. Subtype B was related with transmission clusters with TDR that included 42.6% of the TDR patients. Thanks to resistance testing, 83% of the patients with TDR who started therapy had undetectable viral load whereas half of the patients would likely have received a suboptimal therapy without this test. In conclusion, TDR remained stable and a NNRTI up-and-down trend was observed. While the presence of clusters with TDR is worrying, we could not identify an independent, non-sequence based predictor for TDR or transmission clusters with TDR that could help with guidelines or public health measures.

## Introduction

In recent years, the number of newly diagnosed HIV-1 patients increased in Belgium [1] with a rate of 10.7 per 100,000 population in 2011, one of the highest rates in Europe [2]. Studies carried out in Europe and America highlighted the important role of transmission networks in the spread of transmitted drug resistance (TDR) [3]–[7]. TDR is a clinical and public health issue because it can compromise the response to antiretroviral therapy (ART) at the individual and population level [8]. Three nationwide studies were performed previously in Belgium and reported a TDR prevalence of 29% (67/231; 95% CI: 23.5–35.2) between 1995 and 1998 [9], 7.2% (6/83; 95% CI: 3.4–14.9) in 2000 [10] and 9.5% (27/285, 95% CI: 6.6–13.4) between 2003 and 2006 [11]. However, due to differences in methodology and the lack of a recent study, no up-to-date information is yet available on TDR trends in Belgium. Nevertheless, recent reports revealed the rapid onward transmission of an HIV-1 strain with K103N mutation [12] and the involvement of transmission clusters (TCs) in approximately half of patients with TDR [4] in a local HIV epidemic in Belgium.

Because other studies consistently showed regional differences between the drivers of the HIV-1 epidemic [13], [14], this study aimed to characterize the temporal trend in TDR, the factors associated with TDR including TCs and the clinical impact of TDR for a period of 15 years in a regional epidemic, serviced by the Leuven University Hospitals. The data included socio-demographic, clinical and virological variables.

## Materials and Methods

### Ethics Statement

The research was conducted according to the Declaration of Helsinki. Only patients for whom written informed consent was obtained were included in this study, except patients enrolled in care after 2009. In 2009, UZ Leuven implemented a generic “opt out” system. Patients, who logged an objection to use their medical data for research purposes, were not included in this study. The protocol and this consent procedure were approved by the Ethical Committee UZ Leuven (reference ML-8627, approval B322201316521 S52637).

### Study Population

We analysed data from the cohort of the AIDS Reference Centre (ARC) in Leuven, the capital of the province of Flemish Brabant (Belgium). The ARC in Leuven has been collecting information since 1997 on treated HIV-1 patients and since 1999, also for naive HIV-1 patients, including epidemiological, clinical and virological data, related with the routine patient healthcare services. The prospective clinical use of baseline genotypic drug resistance testing was implemented in 1999 and stored plasma samples from before 1999 were available to retrospectively perform drug resistance testing upon clinician's request. Therefore HIV-1 sequences for drug naive patients were either prospectively or retrospectively obtained from a sample taken at diagnosis, except for 135 patients for whom a later pre-therapy sample was used. The inclusion criteria for the analysis of TDR in the present study were newly HIV-1 diagnosed between January 1998 and December 2012, availability of a nucleotide sequence before antiviral therapy initiation and age older than 18 years, and this cohort was called the Leuven newly-diagnosed (ND) cohort for the purpose of this study. The only exclusion criterion used was documented vertical transmission. Recent infections were defined using clinical and laboratory information such as p24 ELISA, HIV-specific antibody ELISA, and Inno-Lia profile. Patients with the following criteria were classified as recently infected: Fiebig stages I-V [15] or no more than 6 months difference between the last seronegative and first seropositive HIV-1 test [11], CD4 count >200 cells/µl and absence of AIDS-defining conditions [16].

### Drug Resistance Testing

Drug resistance testing was performed using population-based Sanger sequencing of the *pol* gene fragment encoding protease (PR) (amino acids 1 to 99) and 5′-prime end of reverse transcriptase (RT) (amino acids 1 to 320). Sequences were obtained using the ViroSeq HIV-1 Genotyping System version 2 (Celera Diagnostics, Alameda, CA) or with an in-house method upon failure of the commercial test [17]. Sequences with associated information are available through Euresist (http://www.euresist.org).

TDR mutations were defined according to the 2009 list of surveillance drug resistance mutations from the World Health Organization [18]. Therefore, the nucleotide sequences were submitted to the Calibrated Population Resistance tool version 6.0 (http://cpr.stanford.edu/cpr.cgi). The clinical impact of genotypic drug resistance on first line therapy was evaluated using Rega algorithm [19] version 9.1.0 (available at http://rega.kuleuven.be/cev/avd/software/rega-algorithm).

### HIV-1 Subtyping

HIV-1 subtypes and circulating recombinant forms (CRF) were determined using two HIV-1 subtyping tools, namely Rega version 3 (http://www.bioafrica.net/typing-v3/hiv) and COMET version 0.3 (http://comet.retrovirology.lu/) [20]–[22]. Sequences with discordant results were analyzed using manual phylogenetic analysis as was explained previously [22]. Briefly, maximum likelihood (ML) phylogenetic trees under the GTR+Γ nucleotide substitution model were built with RAxML [23] and recombination was verified using SimPlot [24].

### Transmission Cluster Analysis

To investigate the factors associated with TDR and onward transmission of TDR, cluster analyses were performed on the Leuven ND cohort and four additional datasets as controls: (i) all other *pol* sequences from the ARC in Leuven, including treated HIV-1 patients, HIV-1 patients younger than 18 years old and HIV-1 patients with vertical mode of transmission, (ii) HIV-1 *pol* sequences obtained with the search term “Belgium” as sampling country in the Los Alamos HIV sequence database (retrieved from http://www.hiv.lanl.gov date in April 2013), (iii) HIV-1 *pol* sequences from the collaborative study SPREAD that enrolled patients with newly diagnosed HIV-1 infection from 22 European countries including Belgium between 2002 and 2008 (see details of the study in [25], [26]), and (iv) the 30 most similar sequences to the Leuven ND cohort (retrieved by Basic Local Alignment Search Tool (BLAST) from http://blast.ncbi.nlm.nih.gov/Blast.cgi). The quality control of the sequences was performed using the tool available at http://www.hiv.lanl.gov/content/sequence/QC/ and the criteria previously described [27]. Separate datasets were constructed according to subtype. As an out-group, two or three reference sequences of subtype D or B (retrieved from http://www.hiv.lanl.gov) were included for B and non-B subtypes, respectively. Sequences were aligned with Muscle as accessory application in the program Mega version 5 [28], [29]. Duplicates were removed and the positions encoding surveillance drug resistance mutations were excluded [18], which resulted in an average final length of 950 nucleotides. In the resulting dataset, we had 755 sequences for subtype A, 4225 for subtype B, 1036 for subtype C, 202 for subtype F, 665 for subtype G, 440 for CRF01_AE and 677 for CFR02_AG to perform phylogenetic analyses on. Subtypes with frequencies less than 1% were not included in TCs analyses.

A ML tree was inferred with the nucleotide substitution GTR+Γ model and 1000 bootstrap replicates in RAxML [23]. TCs, including pairs (two individuals) and larger clusters (≥3 individuals), were identified by using Cluster Picker (retrieved from http://hiv.bio.ed.ac.uk/software.html) [30] with a genetic distance less or equal than 0.06 substitutions per site and bootstrap support ≥98%. A sensitivity analysis was performed to evaluate the effect of other genetic distances (0.015, 0.030 and 0.045) [31]–[34].

The robustness of the identified TCs was evaluated using Bayesian phylogenetic analysis. TCs and the closest control sequences together with two reference sequences as an out-group were selected and trees were constructed with BEAST v1.7.5 [35] using a lognormal relaxed molecular clock with the SRD06 model of nucleotide substitutions [36] and a Bayesian skyline coalescent prior. The analyses were run in triplicate for 100 million states and trees were sampled every 10000^th^ states. Maximum clade credibility trees (MCC) were summarized using TreeAnnotator after 10% of the burn-in was discarded and visualized with FigTree v.1.4 (available at http://tree.bio.ed.ac.uk). The TCs with a Bayesian posterior probability of 1 were considered robust enough and included in the analysis.

Finally, we defined TCs with TDR as any pair or cluster with more than 3 patients that included at least one patient with TDR from the Leuven ND cohort. The TCs with TDR with more than 3 individuals with similar TDR mutation profile can be suggestive of onward transmission of TDR, they are specifically indicated as TCs with TDR-OT.

### Statistical Analysis

Prevalence of TDR and TDR mutations were calculated with a 95% Wilson score confidence interval (95% CI) on the basis of a binomial distribution, and their trend was calculated by logistic regression analysis. Socio-demographic, virological and clinical variables that were significantly associated with TDR or with TCs with TDR were evaluated in the Leuven cohort. Analyses were performed on patients involved in TCs from the Leuven ND cohort, and from Leuven ND cohort with the other four control datasets. Categorical data were compared using the Chi-square test, the Fisher's exact test or regression techniques as appropriate. The t-test or Mann–Whitney U test was used to compare continuous data. The statistical significance was set at p<0.05 two-sided. All data were analysed using the statistical R software version 2.13.1.

### Bayesian Network Learning

Those factors that were found to be significantly associated with TDR or TCs with TDR in univariate analysis were included in a Bayesian network analysis. This is a probabilistic model that describes statistical conditional dependencies between multiple variables and was performed using the B-course software adapted by Deforche et al [37]. In this analysis, the arcs were scored based on the stability of the conditional dependency assessed with 100 non-parametric bootstrap replicates. The arcs with bootstrap over 75% were considered and depicted in the consensus network.

## Results

### General Characteristics Of The Study Population

778 of the 795 patients who were newly diagnosed with an HIV-1 infection and who received a baseline genotypic drug resistance test between January 1998 and December 2012 at University Hospitals Leuven were included in the analysis, they are referred to as the Leuven ND cohort. Two patients were excluded because their risk group was vertical transmission. For 15 patients, the baseline nucleotide sequence did not fulfill the preset quality criteria: 14 sequences did not have the gene fragments encoding PR or RT, and one sequence was excluded due to the presence of more than four stop codons and indels. The included HIV-1 patients were between 18 and 78 years old and were predominantly male (73.7%), of Belgian origin (58.4%), chronically infected (86.5%) with CDC stage 1 or 2 (67.0%) (Table 1). Patients originating from Belgium were more frequently diagnosed with a recent infection and displayed higher viral loads and CD4 counts (p<0.001). Of all included HIV-1 patients originated from Belgium, 66.7% reported men who have sex with men (MSM) or bisexual contacts as risk factor, whereas 22.5% reported heterosexual contacts. In contrast, HIV-1 patients originating from Sub-Saharan countries reported infection through heterosexual contacts predominantly (79.3%). HIV-1 patients from Sub-Saharan countries were more likely to be co-infected with hepatitis B than patients from Belgium (60.9% vs. 34.8%; OR: 4.49, 95% CI 1.75–12.15, p<0.001). There were 36 HIV-1 therapy-naive patients who did not receive a baseline drug resistance test in this period. This group included more patients of non-Belgian origin (75.0%) and with CD4 count above 500 cells/mL (42.4%, 14/33).

**Table 1:** Characteristics of the Leuven ND cohort and factors associated with TDR.

Characteristics at time of sampling	Total		TDR		Univariate		Multivariate	
				
	n	%	n	%	OR (95% CI)	*p*	OR (95% CI)	*p*
**Patients**	778	100	75	100				
**Male**	573	73.7	67	89.3	3.25 (1.52–7.99)	<0.001*		
**Pregnant women**	15	1.9	1	1.3				
**Age in years at enrolment, Mean (SD)**	37.5 (±10.6)		39.6 (±12.4)					
<25	69	8.9	6	8.0				
25–34	287	36.9	23	30.7				
35–44	241	31.0	23	30.7				
45–54	121	15.6	12	16.0				
>55	60	7.7	11	14.7				
**Country or region of origin**								
Belgium	454	58.4	55	73.3	2.09 (1.20–3.77)	0.006*		
Western Europe (except Belgium)	23	3.0	4	5.3				
High-prevalent regions^†^					0.23 (0.08–0.52)	<0.001*		
Sub-Saharan Africa	198	25.4	7	9.3	0.27 (0.10–0.61)	<0.001*		
Other	24	3.1	0	0				
Other	75	9.6	9	12.0				
Unknown	4	0.5	0	0				
**Risk of transmission**								
MSM	333	42.8	47	62.7	2.44 (1.46–4.15)	<0.001*		
Heterosexual (high-prevalent country)	179	23.0	5	6.7				
Heterosexual (non-endemic)	121	15.6	13	17.3				
Bisexual	32	4.1	5	6.7				
IVDU	14	1.8	1	1.3				
Unknown	75	9.6	1	1.3				
Other	24	3.1	3	4.0				
**Type of infection**								
Chronic	673	86.5	58	77.3				
Recent	105	13.5	17	22.7	2.04 (1.06–3.75)	0.02*		
**CDC stage^§^**								
1 and 2	521	67.0	55	73.3				
3	246	31.6	19	25.3				
Unknown	11	1.4	1	1.3				
**CD4 cell count, median (IQR) ^∥^**	335	(163–493)	365	(236–505)				
<200 cells/mm^3^	228	29.3	16	21.3				
200–349 cells/mm^3^	174	22.4	18	24.0				
350–499 cells/mm^3^	181	23.3	21	28.0				
≥500	182	23.4	19	25.3				
Unknown	13	1.7	1	1.3				
**HIV-RNA load, median (IQR), log copies/ml ^¶^**	4.76	(4.12–5.31)	4.76	(4.16–5.25)				
**Co-infection**								
Hepatitis B	23	3.0	4	5.3				
Negative	412	53.0	36	48.0				
Unknown	343	44.1	35	46.7				
Hepatitis C	406	52.2	1	1.3				
Negative	18	2.3	40	53.3				
Unknown	354	45.5	34	45.3				
**Subtype**								
B	406	52.2	59	78.7	3.77 (2.09–7.17)	<0.001*	3.04 (1.45–6.35)	0.003
**Part of transmission cluster**	226	29.0	32	42.6	1.95 (1.15–3.25)	0.010*		

*Fisher's test or logistic regression techniques were used. ^†^High prevalent countries were defined as HIV-prevalence over 1% in adult population (UNAIDS 2012), ^§^ CDC stage was defined according to 2008 definitions [47], ^∥^CD4 count at diagnosis (median 345, IQR: 158–496) was not statistically different from CD4 count at time of sampling. ^¶^Viral load at diagnosis (median 4.76 IQR: 4.04–5.31) was not statistically different from viral load at time of sampling. Abbreviations: %: percentage, CDC: Center for Disease Control and Prevention, CI: Confidence interval, IVDU: Intravenous drug user, IQR: interquartile range, MSM: men who have sex with men, n: number, OR: odds ratio, SD: standard deviation**.**

The demographic characteristics of the Leuven ND cohort were compared to the general HIV-1 population in Belgium, as reported by the Belgian Scientific Institute of Public Health (information until 2011) (available at www.wiv-isp.be) [1]. The Leuven ND cohort contained more men (73.5% vs. 61.0%, p<0.0002) and Belgians (58.4% vs. 40.6%, p<0.0002) and more MSM (55.9% vs. 42.5%, p<0.0002). National data only covered gender and country of origin from 1998 to 2011, and transmission risk from 2005 to 2011.

### Subtypes

52.2% of the HIV-1 patients were infected with subtype B, followed by CRF02_AG (11.2%), subtype C (10.3%), subtype A (7.7%), CRF01_AE (6.6%), subtype F (2.8%), subtype G (2.1%) and unique recombinant forms (4.8%). Subtypes D, H, J, CRF09_cpx, CRF12_BF, CRF13_cpx, CRF14_BG, CRF18_cpx, CRF22_01A1, CRF37_cpx, and CRF45_cpx were each found in less than 1%. Of the patients with a subtype B infection, 81.0% were of Belgian origin and 71.9% were MSM. Whereas in patients with non-B infections, 33.6% and 51.6% had a Belgian or sub-Saharan origin, respectively, and 69.9% were infected through heterosexual contacts, followed by bisexual/MSM risk factor (13.2%).

### Levels And Trends Of Transmitted Drug Resistance

The overall TDR prevalence was 9.6% (75/778; 95% CI 7.7–11.9). The prevalence of TDR against nucleoside RT inhibitors (NRTI) was 6.5% (51/778; 95% CI 5.0–8.5), against non-NRTI (NNRTI) was 2.2% (17/778; 95% CI 1.4–3.5), and against protease inhibitors (PI) 2.2% (17/778; 95% CI 1.4–3.5). In recently infected individuals, the prevalence of overall TDR was 16.2% (17/105; 95% CI 10.4–24.4), significantly higher than in patients with chronic or unknown duration of infection (8.6%, 58/673; 95% CI 6.7–11.0). The prevalence of TDR by drug class also varied in recently infected individuals. The prevalence of TDR against NRTI was 12.4% (13/105; 95%CI 7.4–20.0), against NNRTI 1.9% (2/105; 95%CI 0.5–6.7), and against PI 6.7% (7/105; 95%CI 3.3–13.1). Dual resistance was detected in 10 patients (1.3%): 3 displayed TDR against NRTI and NNRTI, 6 against NRTI and PI and one against NNRTI and PI. The latter patient with NNRTI and PI resistance and 4 out of 6 individuals with NRTI and PI resistance were recently infected patients (5/105; 4.8%). No triple class resistance was observed.

The majority of the 75 TDR patients displayed one single mutation (70.7%), mainly related to NRTI (58.5%) and NNRTI resistance (24.5%). The revertants at RT position 215 were the most prevalent (44%), followed by M41L (18.7%), K103N (17.3%), L210W (10.7%), K219Q (10.7%), D67N (6.7%), K219R (5.3%), G190A (4.0%), M184V (2.7%), L74V (1.3%), Y115F (1.3%) and Y181C (1.3%). Within PR, I54VT (10.7%) was the most frequent mutation followed by M46IL (9.3%), N88D (6.7%), V82TS (2.7%), L24I (1.3%), I54T (1.3%) and I85V (1.3%). As the inclusion of PR position 46 within the TDR mutation list has been debated due to its polymorphic nature [38], TDR was recalculated excluding this position. This resulted in an overall TDR of 8.9% (69/778; 95% CI 7.0–11.0) and a PI-TDR of 1.4% (11/778; 95% CI 0.8–2.5).

**Figure 1:**
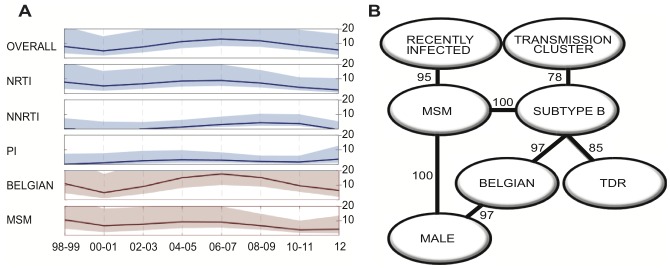
Temporal trends and factors associated with transmitted drug resistance (TDR). (**A**) Trends of prevalence of TDR (percentage) and the 95% confidence intervals (light shading) among newly diagnosed HIV-1 patients at ARC Leuven (Belgium) from 1998 to 2012 are shown for the overall-TDR, NRTI-TDR, NNRTI-TDR, PI-TDR in blue, MSM overall-TDR and Belgian overall-TDR in red. (**B**) The significant variables associated with TDR in the univariate analysis were included in the Bayesian network, the number next to the arcs represents the bootstrap support. Abbreviations: NRTI: nucleoside reverse transcriptase inhibitors, NNRTI: non-nucleoside reverse transcriptase inhibitors, MSM: men who have sex with men, PI: protease inhibitors.

No significant time trends were found in the overall TDR prevalence, nor in transmitted NRTI and PI resistance (see Figure 1A). A parabolic trend was observed for NNRTI-TDR (p = 0.019) with a peak in 2008. That corresponded with a peak in occurrence of K103N (p = 0.026), the only mutation with a temporal trend. Surprisingly, the parabolic temporal NNRTI-TDR trend was not observed in the recently infected individuals. Instead, a stable temporal trend was observed for overall TDR and individual drug classes in this subset of patients. When the analysis was performed according to region of origin, a significant parabolic trend of NNRTI resistance was only found in patients originating from Belgium (p = 0.039).

### Factors Associated With Transmitted Drug Resistance

Univariate analysis was performed to identify predictors of TDR (Table 1), which were male gender (odds ratio (OR) 3.25, 95% CI 1.52–7.99, p<0.001), Belgian origin (OR 2.09, 95% CI 1.20–3.77, p = 0.006), MSM transmission (OR 2.44, 95% CI 1.46–4.15, p<0.001), recent infection (OR 2.04, 95% CI 1.06–3.75, p = 0.02), being part of TCs (OR 1.95, 95% CI 1.15–3.25, p = 0.010) and infected with subtype B virus (OR 3.77, 95% CI 2.09–7.17, p<0.001). Only the latter remained a significant factor (OR 3.04, 95% CI 1.45–6.35, p = 0.003) in multivariate analysis. Since these types of analyses do not display possible interdependencies of the variables and subtype B was most frequently found in MSM originating in Belgium (p<0.001), we evaluated the interdependencies of the variables with a Bayesian network approach. TDR was directly associated only with subtype B, but this subtype was strongly associated with MSM (100% bootstrap support) and with Belgian origin (97% bootstrap support), and to a lesser extent to being part of TCs (78% bootstrap support) (See Figure 1B). To verify whether we could find an important predictor of TDR that could be used in guidelines to target a subpopulation of newly diagnosed for preferential drug resistance testing, we repeated the analysis excluding any information that results from the genotype itself. When subtype B was thus excluded from the analysis, then male gender became directly associated with TDR (64% bootstrap support), and with MSM and Belgian origin (100% bootstrap support), whereas the association between the two latter variables with TCs had lower bootstrap support (31%).

### Transmission Clusters

We identified 114 TCs, 16 of which harbored 32 of the 75 TDR patients from our Leuven ND cohort. Five pairs and eight larger clusters of ≥3 individuals were found among subtype B infected patients, one cluster of 17 individuals with CRF02_AG, and one pair for each subtypes C and CRF01_AE (see Table 2). Six of these 16 TCs with TDR included only a single patient with TDR whereas six clusters were TCs with TDR-OT and included 20 individuals from the Leuven ND cohort (26.7%, 20/75). Singletons were frequently found in patients from the Leuven ND cohort involved in TCs (81.3%, 26/32). Likewise, prevalence against NRTI was the most frequent (81.3%, 26/32), followed by PI (25.0%) and NNRTI (12.5%). Thymidine analogue mutations (TAMs) were predominantly detected in TCs with TDR (81.3%, 26/32), mainly represented by the revertant at position 215 (59.4%, 19/32), followed by the mutations K219QR (18.8%), L210W (15.6%) and M41L (6.3%). Mutations for NNRTI and PI were I54V, N88D (each 15.6%), K103N (12.5%) and M46IL (9.4%).

**Table 2:** Characteristics of transmission clusters containing Leuven patients with TDR.

									TDR mutations		
Cluster	Patient ID^†^	Subtype	Risk of transmission	Country of sampling	Country of origin	Country of infection	Type of infection	Year of diagnosis	NRTI	NNRTI	PI
1	AY165241	B	Unknown	Sweden	Unknown	Unknown	Naive - Unknown	2000*	-	-	-
	***ARCL-1***	***B***	***MSM***	***Belgium***	***Belgium***	***Unknown***	***Naive - Chronic***	***2002***	***M41LM***	-	-
	ESAR-1	B	Heterosexual - Male	Spain	Pakistan	Spain	Naive - Recent	2003	-	-	-
	FJ481828	B	MSM	Spain	Unknown	Unknown	Naive - Unknown	2006*	-	-	-
	ESAR-2	B	MSM	Finland	Russia	Finland	Naive - Chronic	2007	-	-	-
2	EU248399	B	Unknown	Belgium	Unknown	Unknown	Naive - Unknown	2003*	-	K103N	-
	JN101707	B	Unknown	United Kingdom	Unknown	Unknown	Unknown	2004*	-	K103N	-
	***ARCL-2***	***B***	***Heterosexual - Male***	***Belgium***	***Belgium***	***Belgium***	***Naive - Chronic***	***2007***	***-***	***K103N***	***-***
	***ARCL-3***	***B***	***Bisexual - Male***	***Belgium***	***Belgium***	***Unknown***	***Naive - Chronic***	***2009***	***-***	***K103N***	***-***
	JF683797	B	Unknown	Cyprus	Unknown	Unknown	Naive - Unknown	2009*	-	K103N	-
3	***ARCL-4***	***B***	***MSM***	***Belgium***	***Belgium***	***Belgium***	***Naive -Recent*** **^§^**	***2001***	***L210W, T215S***	***-***	***I54V, N88D***
	EU248438	B	Unknown	Belgium	Unknown	Unknown	Naive - Unknown	2003*	L210W, T215S	-	I54V, N88D
	***ARCL-5***	***B***	***MSM***	***Belgium***	***Belgium***	***Belgium***	***Naive - Recent***	***2005***	***L210W, T215S***	***-***	***I54V, N88D***
	***ARCL-6***	***B***	***MSM***	***Belgium***	***Belgium***	***Belgium***	***Naive - Chronic*** **^§^**	***2007***	***L210W, T215S***	***-***	***I54V, N88D***
	***ARCL-7***	***B***	***MSM***	***Belgium***	***Italy***	***Belgium***	***Naive - Recent***	***2010***	***L210W,T215S***	***-***	***I54V,N88D***
	***ARCL-8***	***B***	***MSM***	***Belgium***	***Belgium***	***Unknown***	***Naive - Recent***	***2011***	***L210W, T215S***	***-***	***I54V, N88D***
4	DQ177230	B	Unknown	Belgium	Unknown	Unknown	Naive - Unknown	2002*	T215E	-	-
	***ARCL-9***	***B***	***MSM***	***Belgium***	***Belgium***	***Belgium***	***Naive - Chronic***	***2005***	***T215E***	***-***	***-***
5	***ARCL-10***	***B***	***MSM***	***Belgium***	***Belgium***	***Belgium***	***Naive - Chronic*** **^§^**	***2007***	***T215D***	***-***	***-***
	***ARCL-11***	***B***	***MSM***	***Belgium***	***Belgium***	***Unknown***	***Naive - Chronic***	***2007***	***T215D***	***-***	***-***
	***ARCL-12***	***B***	***MSM***	***Belgium***	***Belgium***	***Unknown***	***Naive - Chronic***	***2007***	***T215D***	***-***	***-***
	***ARCL-13***	***B***	***MSM***	***Belgium***	***Belgium***	***Belgium***	***Naive - Chronic***	***2007***	***T215D***	***-***	***-***
	***ARCL-14***	***B***	***MSM***	***Belgium***	***Belgium***	***Unknown***	***Naive - Chronic***	***2010***	***-***	***-***	***-***
	***ARCL-15***	***B***	***MSM***	***Belgium***	***Belgium***	***Belgium***	***Naive - Chronic***	***2010***	***-***	***-***	***-***
	***ARCL-16***	***B***	***MSM***	***Belgium***	***Belgium***	***Unknown***	***Naive - Recent***	***2011***	***T215D***	***-***	***-***
	***ARCL-17***	***B***	***MSM***	***Belgium***	***Belgium***	***Unknown***	***Naive - Recent***	***2011***	***T215D***	***-***	***-***
	***ARCL-18***	***B***	***MSM***	***Belgium***	***Belgium***	***Unknown***	***Naive - Chronic***	***2012***	***T215D***	***-***	***-***
6	DQ206665	B	Unknown	Argentina	Unknown	Unknown	Naive - Unknown	2004*	-	K103N, P225H	-
	JN670104	B	Unknown	Argentina	Unknown	Unknown	Treated	2005*	M41L, M184V, T215Y	-	D30N, N88D
	***ARCL-19***	***B***	***MSM***	***Belgium***	***Belgium***	***Unknown***	***Naive - Chronic***	***2007***	***T215S***	***K103N***	***-***
	***ARCL-20***	***B***	***MSM***	***Belgium***	***Belgium***	***Belgium***	***Naive - Recent***	***2007***	***-***	***K103N***	***-***
7	***ARCL-21***	***B***	***MSM***	***Belgium***	***Belgium***	***Belgium***	***Naive - Recent***	***2009***	***-***	***-***	***M46L***
	***ARCL-22***	***B***	***MSM***	***Belgium***	***Belgium***	***Unknown***	***Naive - Chronic***	***2010***	***K219Q***	***-***	***-***
	JQ650683	B	MSM	Netherlands	Unknown	Unknown	Unknown	Unknown	-	-	M46L
8	***ARCL-23***	***B***	***MSM***	***Belgium***	***Belgium***	***Unknown***	***Naive - Recent***	***2011***	***T215E***	***-***	***-***
	JQ650714	B	MSM	Netherlands	Unknown	Unknown	Unknown	Unknown	T215E	-	-
9	***ARCL-24***	***B***	***MSM***	***Belgium***	***Belgium***	***Belgium***	***Naive - Chronic***	***2006***	***-***	***-***	***-***
	***ARCL-25***	***B***	***Transfusion - Male***	***Belgium***	***Belgium***	***Unknown***	***Naive - Chronic***	***2010***	***K219RK***	***-***	***-***
10	***ARCL-26***	***B***	***MSM***	***Belgium***	***Indonesia***	***Asia***	***Naive - Chronic***	***2001***	***T215D***	***-***	***-***
	***ARCL-27***	***B***	***MSM***	***Belgium***	***Belgium***	***Belgium***	***Naive - Chronic***	***2003***	***T215D***	***-***	***-***
11	***ARCL-28***	***B***	***MSM***	***Belgium***	***Belgium***	***Unknown***	***Naive - Chronic***	***1998***	***T215C***	***-***	***-***
	***ARCL-29***	***B***	***MSM***	***Belgium***	***Belgium***	***Belgium***	***Naive - Chronic***	***2002***	***T215S***	***-***	***-***
12	***ARCL-30***	***B***	***MSM***	***Belgium***	***Belgium***	***Belgium***	***Naive - Recent***	***2003***	***-***	***-***	***-***
	***ARCL-31***	***B***	***Bisexual - Male***	***Belgium***	***Belgium***	***Belgium***	***Naive - Recent***	***2009***	***K219R***	***-***	***-***
	***ARCL-32***	***B***	***Bisexual - Male***	***Belgium***	***Belgium***	***Unknown***	***Naive - Chronic***	***2009***	***-***	***-***	***-***
	***ARCL-33***	***B***	***Unknown***	***Belgium***	***Belgium***	***Belgium***	***Naive - Chronic***	***2010***	***-***	***-***	***-***
13	EU817049	B	Unknown	United Kingdom	Unknown	Unknown	Naive - Unknown	1998*	K219Q	-	-
	EU817059	B	Unknown	United Kingdom	Unknown	Unknown	Naive - Unknown	2001*	K219Q	-	-
	EU817062	B	Unknown	United Kingdom	Unknown	Unknown	Naive - Unknown	2001*	K219R	-	-
	***ARCL-34***	***B***	***MSM***	***Belgium***	***Belgium***	***Belgium***	***Naive - Chronic***	***2007***	***K219Q***	***-***	***-***
	***ARCL-35***	***B***	***MSM***	***Belgium***	***Belgium***	***Belgium***	***Naive - Recent***	***2006***	***K219Q***	***-***	***-***
	ESAR-1	B	MSM/bisexual	Italy	Italy	Unknown	Naive - Chronic	2003	K219Q	-	-
	EU817050	B	Unknown	United Kingdom	Unknown	Unknown	Naive - Unknown	2003*	K219Q	-	-
	EU817048	B	Unknown	United Kingdom	Unknown	Unknown	Naive - Unknown	2004*	K219R	-	-
	EU817061	B	Unknown	United Kingdom	Unknown	Unknown	Naive - Unknown	2004*	K219Q	-	-
	DQ345509	B	Unknown	Argentina	Unknown	Unknown	Naive - Recent	2005*	-	-	-
	EU817058	B	Unknown	United Kingdom	Unknown	Unknown	Naive - Unknown	2005*	K219Q	-	-
	EU817060	B	Unknown	United Kingdom	Unknown	Unknown	Naive - Unknown	2005*	K219Q	-	-
	EU817065	B	Unknown	United Kingdom	Unknown	Unknown	Naive - Unknown	2005*	K219Q	-	-
	EU817056	B	Unknown	United Kingdom	Unknown	Unknown	Naive - Unknown	2005*	K219Q	-	-
	EU817051	B	Unknown	United Kingdom	Unknown	Unknown	Naive - Unknown	2005*	K219Q	-	-
	EU817068	B	Unknown	United Kingdom	Unknown	Unknown	Naive - Unknown	2005*	K219Q	-	-
	EU817047	B	Unknown	United Kingdom	Unknown	Unknown	Naive - Unknown	2005*	K219Q	-	-
	EU817055	B	Unknown	United Kingdom	Unknown	Unknown	Naive - Unknown	2005*	K219Q	-	-
	EU817063	B	Unknown	United Kingdom	Unknown	Unknown	Naive - Unknown	2005*	K219QR	-	-
	FJ469703	B	Unknown	United States	Unknown	Unknown	Naive - Unknown	2005*	K219Q	-	-
	ESAR-2	B	MSM/bisexual	Germany	Germany	Unknown	Naive - Recent	2006	K219Q	-	-
	ESAR-3	B	MSM/bisexual	Cyprus	Cyprus	Unknown	Naive - Chronic	2007	-	-	-
	ESAR-4	B	MSM/bisexual	Germany	Germany	Germany	Naive - Recent	2007	-	-	-
	JF683765	B	Unknown	Cyprus	Unknown	Unknown	Naive - Unknown	2007*	-	-	-
	JF683791	B	Unknown	Cyprus	Unknown	Unknown	Naive - Unknown	2009*	-	-	-
	JF683808	B	Unknown	Cyprus	Unknown	Unknown	Naive - Unknown	2009*	-	-	-
14	***ARCL-36***	***C***	***Heterosexual - Female***	***Belgium***	***Belgium***	***Unknown***	***Naive - Chronic***	***2012***	***-***	***-***	***M46LM***
	***ARCL-37***	***C***	***Heterosexual - Male***	***Belgium***	***Ethiopia***	***Unknown***	***Naive - Chronic***	***2012***	***-***	***-***	***-***
15	***ARCL-38***	***CRF01_AE***	***Heterosexual - Female***	***Belgium***	***Thailand***	***Thailand***	***Naive - Chronic***	***2005***	***-***	***-***	***-***
	***ARCL-39***	***CRF01_AE***	***Heterosexual - Male***	***Belgium***	***Belgium***	***Belgium***	***Naive - Chronic***	***2005***	***-***	***-***	***M46IM***
16	JX290261	CRF02_AG	Unknown	Russia	Unknown	Unknown	Unknown	2001 or 2002^‡^	-	-	-
	AY829204	CRF02_AG	IVDU - Male	Uzbekistan	Unknown	Unknown	Unknown	2002*	-	-	-
	AY829207	CRF02_AG	IVDU - Male	Uzbekistan	Unknown	Unknown	Unknown	2002*	-	-	-
	AY829214	CRF02_AG	IVDU - Male	Uzbekistan	Unknown	Unknown	Unknown	2002*	-	-	-
	HQ449394	CRF02_AG	Unknown - Female	Russia	Unknown	Unknown	Unknown	2005^‡^	-	-	-
	DQ465230	CRF02_AG	MTCT	USA	Unknown	Unknown	Unknown	2006*	-	-	-
	GQ290726	CRF02_AG	Unknown	South Korea	Unknown	Unknown	Naive - Unknown	2008*	-	-	-
	GQ290743	CRF02_AG	Unknown	South Korea	Unknown	Unknown	Naive - Unknown	2008*	-	-	-
	HQ412530	CRF02_AG	Unknown - Male	Russia	Unknown	Unknown	Unknown	2008*	L74I, M184V, K219E	L100I, K101E, Y181C, G190S	-
	HQ115069	CRF02_AG	Unknown - Male	Ukraine	Unknown	Unknown	Unknown	2009^‡^	-	-	-
	JX500703	CRF02_AG	Unknown	Russia	Unknown	Unknown	Unknown	2010*	-	-	-
	***ARCL-40***	***CRF02_AG***	***Heterosexual - Female***	***Belgium***	***Kazakhstan***	***Unknown***	***Naive - Chronic***	***2011***	***K219RK***	***-***	***-***
	JX500697	CRF02_AG	Unknown	Russia	Unknown	Unknown	Unknown	2011*	-	-	-
	JX500706	CRF02_AG	Unknown	Russia	Unknown	Unknown	Unknown	2011*	-	-	-
	KC509858	CRF02_AG	Unknown - Female	Russia	Unknown	Unknown	Unknown	2012*	-	-	-
	KC120872	CRF02_AG	Unknown	South Korea	Unknown	Unknown	Unknown	Unknown	-	-	-
	KC120881	CRF02_AG	Unknown	South Korea	Unknown	Unknown	Unknown	Unknown	-	-	-

Abbreviations: ARCL: AIDS Reference Center Leuven, CRF: Circulating recombinant form, ESAR: European Society for Translational Antiviral Research, IVDU: intravenous drug user, NRTI: nucleoside reverse transcriptase inhibitors, NNRTI: non-nucleoside reverse transcriptase inhibitors, MSM: men who have sex with men, MTCT: mother to child transmission, PI: protease inhibitors,^†^Patient ID includes patients of the Leuven cohort (bold and italics), ESAR controls and accession numbers of NCBI database *Control sequences have available year of sampling. ^‡^Control sequences with year of diagnosis available. ^§^Sequences were also included when the patient was on antiretroviral treatment.

The characteristics of the Leuven ND cohort patients involved in TCs were evaluated. Patients carrying TDR were significantly more associated with TCs compared to patients without TDR (OR: 1.95, see Table 1 and Table S1) and the association remained when considering only larger clusters by excluding pairs (OR: 2.86, 95% CI 1.59–5.01, p = <0.001). Similarly, when including only TCs with TDR-OT, TDR remained significantly associated with TCs (OR: 2.44, 95% CI 1.32–4.36, p = 0.002, see Table S1). 32 out of 75 TDR patients were involved in TCs (42.6%, 95% CI 32.1–53.9), while of the 703 patients without TDR, only 194 were found in TCs (27.6%, 95% CI 24.4–31.0). As expected, Leuven ND cohort patients with TDR and involved in TCs were significantly more of Belgian origin (90.6% versus 65.5%; OR: 4.84, 95% CI 1.41–25.78, p = 0.005), infected with subtype B (90.6% versus 63.9%; OR: 5.42, 95% CI 1.59–28.85, p = 0.001) and characterized with MSM risk factor (78.1% versus 52.6%; OR: 2.71, 95% CI 1.07–7.84, p = 0.03) than their counterparts without TDR (see Table S1). When we focussed on predictors for TCs with TDR by including the data of controls, subtype B remained significantly associated with TCs with TDR (77.4% versus 65.1%, OR: 1.83 95% CI 1.03–3.35, p = 0.036). However, TCs of patients with TDR were larger than TCs without TDR (median 3.5 vs. 2 patients per TC, OR: 1.43 95% CI 1.08–1.91, p = 0.001). Belgium as sampling country or as country of origin and heterosexual contact were then more frequent in the group of TCs that included solely patients without evidence of TDR. Multivariate analysis did not show any significant factor associated with patients in TCs with TDR versus other TCs. Finally, we also performed separate analyses on TCs with TDR-OT (see Table S1). The same variables remained significantly associated with TCs in the univariate analysis, with the exception of recent infection that became significant. The median of the TCs with TDR-OT was larger than TCs without TDR (5.5 versus 2 patients per TCs, OR: 2.01 (1.29–3.11). Likewise, none of the variables were significant in the multivariate analysis.

**Figure 2:**
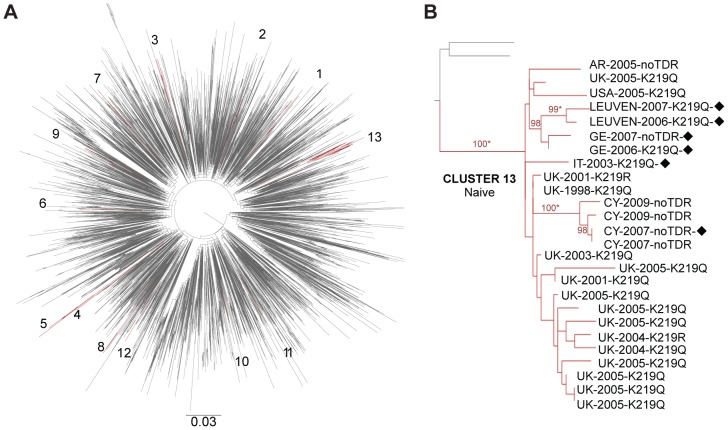
Examples of subtype B transmission clusters (TCs) with TDR: A maximum likelihood (ML) tree per subtype was constructed, and TCs were confirmed by Bayesian Phylogenetic analyses. (**A**) The ML tree for subtype B (Leuven ND cohort and control sequences) with the TCs colored in dark red. (**B**) The largest TC of subtype B: composed of therapy-naive patients, several nationalities and mutations at RT position 219; bootstrap values above 98% are shown. Abbreviations: AR: Argentina, CY: Cyprus, GE: Germany, IT: Italy, UK: United Kingdom, USA: United States of America, black diamond: men who have sex with men, asterisk: posterior distribution equal to 1 in the Bayesian phylogenetic analysis.

The characteristics of the Leuven TDR patients involved in TCs are shown in Table 2. Five pairs included mainly naive MSM originating from Belgium, infected with a subtype B strain carrying one of the 215 revertants, whereas two pairs with subtype C and CRF01_AE strains displaying mutations at PR position 46 included heterosexual Belgians with a foreign partner. Two large subtype B TCs with TDR included 9 or more patients. Cluster number 5 was composed of nine therapy-naive individuals originating from Belgium with MSM as a risk factor and diagnosed between 2007 and 2012. Seven of them displayed a revertant at RT position 215 while two strains had no TDR. Cluster number 13 (Figure 2B) included 26 naive patients mainly from United Kingdom and countries of western and southern Europe, western Asia and America. The main mode of transmission was MSM infected with viruses carrying mutations at position 219, except for six individuals who mainly originated from Cyprus and did not display any mutations. The remaining subtype B TCs were composed of three to six individuals. All had at least one individual originating from Belgium and one from another country. For instance, cluster number 1 included one patient originating from Belgium and two patients from Russia and Pakistan but infected in other countries like Finland and Spain. One peculiar subtype B cluster with extensive NRTI and PI resistance (cluster number 3) contained 6 therapy-naive MSM with TDR all with a Belgian connection, either infected in Belgium or originating from Belgium. On the other hand, the largest cluster of non-B subtypes was composed of 17 individuals infected with CRF02_AG and sampled in different countries from Central-eastern Asia and Eastern Europe and characterized by different risks factors including heterosexual orientation, intravenous drug user (IVDU) or vertical transmission. The resistance pattern was also heterogeneous in this cluster. The majority of patients did not display TDR mutations, whereas one naive patient sampled in Belgium and originating from Kazakhstan displayed a mixture of K219RK, and one patient who was tested in Russia was probably treated and displayed high-level resistance against NRTI and NNRTI.

Since the definition of clustering is still a matter of debate, additional analyses were performed to assess the impact of the genetic distance on the identification of TCs with values of 0.015, 0.03 and 0.045. As a result, the number of TCs with TDR decreased to 9, 13 and 17, respectively, including 22.7%, 29.3% and 36.0% of the 75 patients with TDR. The number of TCs that included TDR patients was larger with the genetic distance 0.045 because the cluster with 9 individuals identified as number 5 in Table 2 was split in one pair (ARCL-16-TDR and ARCL-18-TDR) and one cluster with the remaining patients. With the stringent criteria of 0.015, TDR was still associated with TCs (OR: 2.56 05% CI 1.32–4.76, p = 0.003). Subtype B infection remained as the factor associated with TCs with TDR (p = 0.002), whereas sociodemographic factors such as Belgian nationality and MSM contact were not significantly linked.

### Potential Impact Of TDR On First-Line Regimen

For each patient with TDR, we analysed the genotypic susceptibility score (GSS) of the three regimens most frequently prescribed during the year the patient was diagnosed, and for each patient who in the meantime had started treatment we also estimated the GSS of the first-line therapy received. Rega algorithm 9.1.0 was used for these analyses, which has a recommended GSS for patients with TDR. This recommendation was introduced in March 2007 and suggests a regimen with GSS of ≥3.5 when TDR is detected, thereby suggesting a triple therapy with a fully active boosted PI which receives a score 1.5, but not with an NNRTI which receives a score of 1 when fully active.

Amongst the 636 patients who started ART, the GSS of the actual prescribed first-line regimen was ≥3.5 in 261 patients (41.0%), 3 in 357 patients (56.1%), and <3 in 18 patients (2.8%). The latter group included five patients without TDR, of which three patients were treated with bi-therapy in 1998, one with bi-therapy including a PI in 2002 and one with a mono PI regimen in 2010. Although the demographic characteristics were similar between the patients with a GSS ≥3 and <3, the latter group more often started ART before 2002 (8/18, p<0.001).

In the group of patients with viruses carrying TDR, 60 started therapy before the end of 2012. The GSS was ≥3.5 in 34 patients (56.7%), equal to 3 in 13 patients (21.7%), and <3 in 13 patients (21.7%). The latter group displayed resistance only to NRTIs (6/13), only to PI (2/13) or dual resistance (5/13).

Sustained undetectable viral load during the first-line therapy was reached in 83.3% (50/60) of the TDR patients, whereas 3.3% of the patients (2/60) had sustained low level viremia without evidence of virological rebound above 500 copies/ml. Four patients (6.7%) had early changes in ART due to toxicity and 1 patient (1.7%) died shortly after therapy initiation. Only 5.1% of the patients (3/60) displayed virological failure and the emergence of major NNRTI resistance-related mutations. Two of these patients displayed evidence of only NRTI TDR mutations at baseline and received a NRTI+NNRTI regimen with a GSS equal to 3. The third patient started a NRTI+NNRTI therapy with a GSS equal to 1 in 2004, two days after the first contact date and two weeks before the drug resistance results were available. In this patient, the therapy was quickly changed after receiving the baseline drug resistance report indicating NRTI and NNRTI TDR mutations and observing no virological response. Subsequent drug resistance testing on a later sample revealed further accumulation of NNRTI resistance.

To appreciate the value of baseline drug resistance testing, the GSS was calculated for the three most frequently prescribed first-line regimens in patients displaying TDR in our cohort per year (see Table S2). Theoretically, 49.3% of TDR patients (37/75) were likely to receive a potential suboptimal regimen (GSS<3) if baseline drug resistance testing had not been available to the treating physician. However, the frequency of these patients is decreasing over time (p = 0.002). Up until 2003, the most common first-line regimens included a thymidine analogue with either a NNRTI- or unboosted PI. Thereafter, tenofovir or abacavir were more commonly used as supporting NRTI, but a GSS<3 was still mainly observed for NRTI+NNRTI regimens. A low GSS under a boosted PI based regimen would only have accounted for approximately 15% of the TDR cases per year between 2005 and 2011.

## Discussion

The TDR surveillance in the 778 included patients who were newly diagnosed with HIV-1 at our clinic in Leuven showed a stable overall prevalence of 9.6% between 1998 and 2012. This result is in line with the 9.5% of the latest national survey that included 285 patients who were newly diagnosed in Belgium between 2003 and 2006 [11]. It was also consistent with the stable overall TDR levels of 9.7% in Spain and of 9.0% in France, results from national surveys with a similar design and time frame as our study [34], [39], and with the overall trend of 8.9% in Europe between 2002 and 2007 [26], [40]. However, the overall TDR in our local epidemic was higher than the 5.6% between 2003 and 2010 in Sweden [5] and the 6.5% between 2001 and 2009 in Ghent (Belgium) [4]. These regional differences highlight the importance of studying local epidemics and suggest that TDR prevalence may vary within a single country. Indeed, our findings may not be generalizable to the HIV-1 epidemic in Belgium because our cohort has a higher prevalence of MSM and of individuals originating from Belgium. It should be pointed out, however, that the demographic characteristics data of our study population was more complete than the national database for which nationality and mode of transmission were not available in approximately 25% of the cases. Although our study could have overestimated the level of TDR, due to patients who were unwilling to disclose their ART status, measures were taken to decrease the number of misclassifications. Patient records were exhaustively revised by clinicians and virologists, and individuals with evidence of drug resistance and viral load profiles suggestive of treatment were not considered drug naive.

While subtype B, being MSM, male gender, originating from Belgium, recently infected, and involvement in TCs were all significant predictors for TDR, only subtype B remained significantly associated in the multivariate analysis. Bayesian analyses however, showed the dependency of this factor on being of Belgian origin and MSM. Similarly when subtype B was excluded, male gender became directly associated with TDR with low support but significantly dependent on Belgian origin and MSM. These findings are in agreement with previous reports from other Belgian, European and American studies [4], [5], [11], [39], [41]. In a recent study, the association of subtype with country of sampling, risk group and gender has been interpreted as evidence for highly compartmentalized epidemics in Europe [42]. Therefore, the early introduction of subtype B in European MSM and their broad access to HIV care and ART for decades might explain the single direct association of subtype B with TDR in many resource-rich settings. Nevertheless, recent studies revealed an increasing prevalence of TDR among Sub-Saharan African migrants residing in Spain and Sweden, potentially linked to the increasing drug resistance levels in Africa [5], [43]. However in our cohort, Sub-Saharan African patients were still associated with less TDR and we did not observe a time trend in those patients (data not shown).

Fluctuations in TDR levels were observed over the entire study period, but the only significant trend was a parabolic trend detected for transmitted NNRTI resistance. The overall NNRTI TDR prevalence was 2.2%, with a maximum of 6.5% in 2008-2009. This parabolic trend, mainly linked to the detection of K103N and to a Belgian origin, was not observed among recently infected patients. Although a parabolic trend with a peak in 2004 was also described in the SPREAD study that included data up until 2005 [26], the same surveillance up to 2007 showed a linear increase over time [40] potentially associated with the frequent use of NNRTI in first-line regimens as the authors suggested. Similarly, the local change of prescribing practices to more potent regimens in later years, use of drug resistance testing and the longer time period analyzed in this study could explain the parabolic trend. In the total cohort, TDR associated with NRTI and PI resistance fluctuated around 6.5% and 2.2%, respectively. Among recently infected patients, NRTI- and PI-TDR levels increased to 12.4% and 6.7% respectively. This increase was not observed for NNRTI resistance, presumably due to the lower impact of NNRTI mutations on viral fitness with consequently a lower likelihood of reversion to wild-type and of a TDR underestimation by population-based Sanger sequencing in chronically infected patients.

Singletons were predominantly detected in our cohort, with TAMs as the most commonly observed. Although the use of zidovudine has decreased over the last few years, we did not find any time trend for TAMs. The peak in NNRTI TDR in 2008 was not related to clustered transmission of TDR or migration from other countries as has been suggested in other settings [12], [43]. However, it might have been linked with the enhanced use of NNRTI-containing combinations in the years before. From 2009 onwards, the most commonly prescribed regimen was the potent combination tenofovir+emtricitabine+efavirenz. Up until 2012, no other available NNRTI- or PI/ritonavir-based regimen had proven superior to this regimen with respect to virologic responses.

If resistance testing had not been performed and patients with TDR would have received one of the preferred first-line regimens at that time, approximately half of them would likely have received a regimen in which the virus had lost susceptibility to at least one of the prescribed drugs. Irrespective of the detected TDR by population-based Sanger sequencing, all of them would have had a higher risk of virological failure, as NNRTI-based regimes were commonly prescribed from 2002 onwards [8]. However, 83% of the patients with TDR and who started ART achieved undetectable viral load thanks to the prescription of potent regimens enabled by the availability of drug resistance results. Only 3 patients with baseline resistance to NRTI had virological failure with development of NNRTI resistance after the initiation of a NRTI+NNRTI regimen.

In this cohort, 42.6% of the TDR patients were involved in TCs, which included nine clusters and seven pairs. Because we were interested in observing TCs over a period of 15 years, a genetic distance of 0.06 substitutions per site and a bootstrap support of ≥98% were used to define TCs. These TCs were also confirmed using Bayesian phylogenetic techniques, indicating that the obtained results were robust [4], [7], [34]. Although, the comparison between studies of transmission networks is difficult due to the differences in sampling, phylogenetic techniques and the lack of a standardized TC definition, our results were in line with a study carried out in Ghent (Belgium) in which 18 out of 33 (55%) TDR patients were involved in pairs or larger clusters [4]. When a more stringent criterion of 0.015 genetic distance was used, the percentage of TDR patients involved in TCs decreased to approximately 23%. In general, some conclusions can be drawn from the TCs analyses. First, none of the factors were significantly associated with TCs with TDR in the multivariate model, although a dependency between subtype B and clustering was found in the Bayesian network and TDR was significantly associated with TCs in the univariate analysis. Therefore we were unable to identify a non-sequence based predictor of being in TCs with TDR, even though the odds were higher for patients who were MSM and originating from Belgium. This result is similar to other studies performed in Europe [4], [34]. Second, TAMs were more frequently found in TCs and this was also observed in the Ghent cohort and other settings [3], [4], [7]. Third, TCs with TDR involved mainly therapy-naive individuals, chronically or recently infected, rather than ART-experienced patients, which could suggest that drug naive people, potentially unaware of their HIV seropositive status, are the main source of TDR instead of patients failing ART [3], [6], [7]. Fourth, 7 out of 16 TCs with TDR involved patients of different nationalities. Although, we were not able to retrace the country of infection in many instances, this may imply that migration plays an important role in the local spread of subtype B as previously described [44], but also of TDR. Fifth, spread of non-B subtypes in the local epidemic was still limited and was related with heterosexuals as has been described in other epidemics [6]. They were also not prone to spread TDR. They often involved Belgians and other nationalities that could imply a limited intermixing of the HIV-1 epidemic between locals and immigrants.

Although we used all the sequences available in public databases and from a collaborative European dataset, for 7 TCs with TDR we did not find evidence that patients other than the ones followed at our clinic were involved in transmission networks. Similarly, 6 TCs with TDR patients from the Leuven ND cohort included only a single patient with TDR, and for these patients, no evidence of onward transmission of TDR is available. When 3 or more patients in a TC had the same TDR mutation profile, we indicated the cluster as TDR-OT, since this is suggestive of onward transmission of TDR, although we cannot exclude that all these TDR patients received their resistance from a treated patient. The association between TDR and TCs remained, also for those TCs with TDR-OT. Since 27% of the patients from the Leuven ND cohort were involved in those TCs, this could imply an important role of local transmission on the spread of TDR. Nevertheless, we cannot exclude the possibility that the networks might include other intermediary individuals who were not sampled or unaware of their seropositive status, known limitations of phylogenetic analyses.

Singletons and the TAM 215 were predominant in TCs, but the clinical impact on the current first-line therapies remains limited. However, two TCs that involved MSM individuals originating from Belgium with viruses carrying the K103N were detected. The latest diagnosis date was 2009 in these TCs, suggesting that in our local epidemic this mutation was not involved in a recent spread in contrast to a reported outbreak in Namur (Belgium) [12]. Continuous monitoring of the spread of this mutation is required to establish the impact on current practices. On the other hand, two large clusters were detected with the TAM 219 and they involved different nationalities from Europe, Asia and America. One of these TCs contained one patient living in Belgium but originating from Kazakhstan, and control sequences from Uzbekistan that were part of an outbreak of CRF02_AG among IVDU [45]. Our analyses revealed the involvement of other countries and risk groups and the absence of K219R in many of the clustered sequences. The other large cluster included MSM individuals infected with subtype B and control sequences mainly from United Kingdom [46] and other countries in Europe. The majority of strains in this cluster displayed K219Q with only a few strains displaying K219R or no TDR.

In summary, this study showed a stable trend of almost 10% overall TDR between 1998 and 2012 in our Leuven (Belgium) cohort. TDR associated with NNRTI resistance displayed a parabolic trend that overlapped with an up-and-down NNRTI TDR trend in Belgians and with the trend of K103N. Our cohort was mainly composed of chronically infected patients and around 43% of the patients with TDR were involved in transmission networks, suggesting public health policies that target early diagnoses of recently infected patients are needed. Although the main factor related with TDR was subtype B, this variable was dependent on Belgian nationality and MSM mode of transmission. While these variables were also associated with being in TCs with TDR, we were unable to significantly identify a population that could be targeted for future TDR prevention policies. More local, national and international surveillance studies are needed to confirm the significance and durability of our observations, as changes in TDR levels and patterns are not straightforward to predict due to potential changes in prevention, testing and treatment strategies and changes in other potentially important drivers, such as e.g. behaviour and migration.

## Supporting Information

Table S1 **Characteristics of patients from the Leuven ND cohort and from patients involved in transmission clusters.** Transmission clusters with likely onward transmission included clusters number 2, 3, 5, 6, 7, 13 in Table 2. Multivariate analysis was not significant in any of the analyses. Abbreviations: CI confidence intervals, IVDU intravenous drug user, MSM men who have sex with men, n sample, OR odds ratio, % percentage
(DOC)

Table S2 **Impact of transmitted drug resistance (TDR) on clinical care: The genotypic susceptibility score (GSS) of each sequence with TDR was calculated for the antiretroviral regimens most frequently prescribed in the year of diagnosis (top three).** For instance, the GSS was less than 3 for each of the most frequently prescribed regimens in the only sequence with TDR sampled in 1998. According to the Rega algorithm, a GSS of at least 3.5 is advised for the first-line therapy in a patient carrying a virus with TDR. Abbreviations: ART antiretroviral therapy, 3TC lamivudine, ABC abacavir, ATV atazanavir, ATV/r ritonavir-boosted atazanavir, AZT zidovudine, D4T stavudine, DDI didanosine, DRV/r ritonavir-boosted darunavir, EFV efavirenz, FPV/r ritonavir-boosted fosamprenavir, FTC emtricitabine, IDV indinavir, LPV/r ritonavir-boosted lopinavir, NFV nelfinavir, NVP nevirapine, TDF tenofovir disoproxil fumarate.
(DOCX)
