# Supplementary material for: Trends and Predictors of Transmitted Drug Resistance (TDR) and Clusters with TDR in a Local Belgian HIV-1 Epidemic
Source: PLoS One. 2014 Jul 8;9(7):e101738. doi: 10.1371/journal.pone.0101738 (PMC4086934; doi:10.1371/journal.pone.0101738)
Supplement: Table S1 — Characteristics of patients from the Leuven ND cohort and from patients involved in transmission clusters. Transmission clusters with likely onward transmission included clusters number 2, 3, 5, 6, 7, 13 in Table 2. Multivariate analysis was not significant in any of the analyses. Abbreviations: CI confidence intervals, IVDU intravenous drug user, MSM men who have sex with men, n sample, OR odds ratio, % percentage (DOC) [file pone.0101738.s001.doc]

**Table S1:** **Characteristics of patients from the Leuven ND cohort and** **from patients involved in transmission clusters.** Transmission clusters with likely onward transmission included clusters number 2, 3, 5, 6, 7, 13 in Table 2. Multivariate analysis was not significant in any of the analyses. Abbreviations: CI confidence intervals, IVDU intravenous drug user, MSM men who have sex with men, n sample, OR odds ratio, % percentage

| **Characteristic** | **All clusters** | | | | | | | | | | | | | | | | **Clusters with likely onward transmission** | | | | | | | |
| --- | --- | --- | --- | --- | --- | --- | --- | --- | --- | --- | --- | --- | --- | --- | --- | --- | --- | --- | --- | --- | --- | --- | --- | --- |
|  | **Leuven ND cohort** | | | | | | | | **Leuven ND cohort and controls** | | | | | | | | **Leuven ND cohort** | | | | **Leuven ND cohort and controls** | | | |
|  | **Total** | | **Patients** | | **Patients** | | **Univariate** | | **Total** | | **Clusters** | | **Clusters** | | **Univariate** | | **Clusters** | | **Univariate** | | **Clusters** | | **Univariate** | |
|  |  |  | **with TDR** | | **without TDR** | |  |  |  |  | **with TDR** | | **without TDR** | |  |  | **with TDR** | |  |  | **with TDR** | |  |  |
|  | **n** | **%** | **n** | **%** | **n** | **%** | **OR** | **p value** | **n** | **%** | **n** | **%** | **n** | **%** | **OR** | **p value** | **n** | **%** | **OR** | **p value** | **n** | **%** | **OR** | **p value** |
|  |  |  |  |  |  |  | **(95% CI)** |  |  |  |  |  |  |  | **(95% CI)** |  |  |  | **(95% CI)** |  |  |  | **(95% CI)** |  |
| Number of patients | 226 | 100 | 32 | 100 | 194 | 100 | 1.95 | 0.01 | 351 | 100 | 93 | 100 | 258 | 100 |  |  | 20 |  | 2.44 |  | 53 |  |  |  |
|  |  |  |  |  |  |  | (1.15-3.25) |  |  |  |  |  |  |  |  |  |  | 100 | (1.32-4.36) | 0.002 |  | 100 |  |  |
| Number of clusters | 114 | 100 | 16 | 100 | 98 | 100 |  |  | 114 | 100 | 16 | 100 | 98 | 100 |  |  | 6 | 100 |  |  | 6 | 100 |  |  |
| Median of patients per cluster (IQR) | - | - | - | - | - | - |  |  | 3 | (2-3) | 3.5 | (2-5.2) | 2 | (2-3) | 1.43 | 0.001 | - | - |  |  | 5.5 | (4.2-8.2) | 2.01 |  |
|  |  |  |  |  |  |  |  |  |  |  |  |  |  |  | (1.08-1.91) |  |  |  |  |  |  |  | (1.29-3.11) | <0.0001 |
| Gender |  |  |  |  |  |  |  |  |  |  |  |  |  |  |  |  |  |  |  |  |  |  |  |  |
| Male | 187 | 82.7 | 30 | 93.8 | 157 | 80.9 |  |  | 233 | 66.4 | 51 | 54.8 | 182 | 70.5 |  |  | 20 | 100 |  |  | 29 | 54.7 |  | <0.0001 |
| Female | 39 | 17.3 | 2 | 6.3 | 37 | 19.1 |  |  | 47 | 13.4 | 5 | 5.4 | 42 | 16.3 |  |  | - | - |  |  | - | - |  |  |
| Missing |  |  |  |  |  |  |  |  | 71 | 20.2 | 37 | 39.8 | 34 | 13.2 |  |  | - | - |  |  | 24 | 45.3 |  |  |
| Type of Infection |  |  |  |  |  |  |  |  |  |  |  |  |  |  |  |  |  |  |  |  |  |  |  |  |
| Naive | 226 | 100 | 32 | 100 | 194 | 100 |  |  | 308 | 87.7 | 74 | 79.6 | 234 | 90.7 |  |  | 20 | 100 |  |  | 50 | 94.3 |  |  |
| Recent | 46 | 20.4 | 11 | 34.4 | 35 | 18 |  |  | 57 | 16.2 | 16 | 17.2 | 41 | 15.9 |  |  | 9 | 45 |  | 0.023 | 12 | 22.6 |  | <0.0001 |
| Main risk of transmission |  |  |  |  |  |  |  |  |  |  |  |  |  |  |  |  |  |  |  |  |  |  |  |  |
| MSM | 127 | 56.2 | 25 | 78.1 | 102 | 52.6 | 2.71 | 0.03 | 178 | 50.7 | 41 | 44.1 | 137 | 53.1 |  |  | 18 |  |  |  |  |  |  |  |
|  |  |  |  |  |  |  | (1.07 - 7.84) |  |  |  |  |  |  |  |  |  |  | 90 |  | 0.016 | 28 | 52.8 |  |  |
| Heterosexual | 64 | 28.3 | 4 | 12.5 | 60 | 30.9 |  |  | 81 | 23.1 | 7 | 7.5 | 74 | 28.7 | 0.29 | 0.002 |  |  |  |  |  |  |  |  |
|  |  |  |  |  |  |  |  |  |  |  |  |  |  |  | (0.10-0.70) |  | 1 | 5 |  |  | 1 | 1.9 |  | <0.0001 |
| IVDU | 5 | 2.2 | 0 | 0 | 5 | 2.6 |  |  | 8 | 2.3 | 3 | 3.2 | 5 | 1.9 |  |  | - | - |  |  | - | - |  |  |
| Others | 16 | 7.1 | 3 | 9.4 | 13 | 6.7 |  |  | 3 | 0.9 | 2 | 2.2 | 1 | 0.4 |  |  | 1 | 5 |  |  | - | - |  |  |
| Missing | 14 | 6.2 | 0 | 0 | 14 | 7.2 |  |  | 81 | 23.1 | 40 | 43 | 41 | 15.9 |  |  | - | - |  |  | 24 | 45.3 |  |  |
| Country of origin |  |  |  |  |  |  |  |  |  |  |  |  |  |  |  |  |  |  |  |  |  |  |  |  |
| Belgium | 156 | 69 | 29 | 90.6 | 127 | 65.5 | 4.84 | 0.005 | 160 | 45.6 | 35 | 37.6 | 125 | 48.4 |  |  |  |  |  |  |  |  |  |  |
|  |  |  |  |  |  |  | (1.41 - 25.78) |  |  |  |  |  |  |  |  |  | 19 | 95 |  | 0.039 | 21 | 39.6 |  |  |
| High-prevalent country | 39 | 17.3 | 0 | 0 | 39 | 20.1 |  |  | 43 | 12.3 | 2 | 2.2 | 41 | 15.9 |  |  | - | - |  |  | - | - |  |  |
| Other | 29 | 12.8 | 3 | 9.4 | 26 | 13.4 |  |  | 52 | 14.8 | 9 | 9.7 | 43 | 16.7 |  |  | 1 | 5 |  |  | 5 | 9.4 |  |  |
| Missing | 2 | 0.9 | 0 | 0 | 2 | 1 |  |  | 96 | 27.4 | 47 | 50.5 | 49 | 19 |  |  | - | - |  |  | 27 | 50.9 |  |  |
| Sampling country |  |  |  |  |  |  |  |  |  |  |  |  |  |  |  |  |  |  |  |  |  |  |  |  |
| Belgium | 226 | 100 | 32 | 100 | 194 | 100 |  |  | 247 | 70.4 | 43 | 46.2 | 204 | 79.1 | 0.22 | <0.0001 | 20 |  |  |  |  |  |  | <0.0001 |
|  |  |  |  |  |  |  |  |  |  |  |  |  |  |  | (0.13-0.39) |  |  | 100 |  |  | 24 | 45.3 |  |  |
| Other countries | - | - | - | - | - | - |  |  | 104 | 29.6 | 50 | 53.8 | 54 | 20.9 |  |  | - | - |  |  | 29 | 54.7 |  |  |
| Subtype |  |  |  |  |  |  |  |  |  |  |  |  |  |  |  |  |  |  |  |  |  |  |  |  |
| A | 13 | 5.8 | 0 | 0 | 13 | 6.7 |  |  | 20 | 5.7 | 0 | 0 | 20 | 7.8 |  |  | - | - |  |  | - | - |  |  |
| B | 153 | 67.7 | 29 | 90.6 | 124 | 63.9 | 5.42 | 0.001 | 240 | 68.4 | 72 | 77.4 | 168 | 65.1 | 1.83 | 0.036 | 20 |  |  |  |  |  |  |  |
|  |  |  |  |  |  |  | (1.59 - 28.85) |  |  |  |  |  |  |  | (1.03-3.35) |  |  | 100 |  | 0.011 | 53 | 100 |  | <0.0001 |
| C | 18 | 8 | 1 | 3.1 | 17 | 8.8 |  |  | 22 | 6.3 | 2 | 2.2 | 20 | 7.8 |  |  | - | - |  |  | - | - |  |  |
| F | 1 | 0.4 | 0 | 0 | 1 | 0.5 |  |  | 3 | 0.9 | 0 | 0 | 3 | 1.2 |  |  | - | - |  |  | - | - |  |  |
| G | 3 | 1.3 | 0 | 0 | 3 | 1.5 |  |  | 4 | 1.1 | 0 | 0 | 4 | 1.6 |  |  | - | - |  |  | - | - |  |  |
| CRF01_AE | 13 | 5.8 | 1 | 3.1 | 12 | 6.2 |  |  | 16 | 4.6 | 2 | 2.2 | 14 | 5.4 |  |  | - | - |  |  | - | - |  |  |
| CRF02_AG | 25 | 11.1 | 1 | 3.1 | 24 | 12.4 |  |  | 46 | 13.1 | 17 | 18.3 | 29 | 11.2 |  |  | - | - |  |  | - | - |  |  |
