# Supplementary material for: Trends and Predictors of Transmitted Drug Resistance (TDR) and Clusters with TDR in a Local Belgian HIV-1 Epidemic
Source: PLoS One. 2014 Jul 8;9(7):e101738. doi: 10.1371/journal.pone.0101738 (PMC4086934; doi:10.1371/journal.pone.0101738)
Supplement: Table S2 — Impact of transmitted drug resistance (TDR) on clinical care: The genotypic susceptibility score (GSS) of each sequence with TDR was calculated for the antiretroviral regimens most frequently prescribed in the year of diagnosis (top three). For instance, the GSS was less than 3 for each of the most frequently prescribed regimens in the only sequence with TDR sampled in 1998. According to the Rega algorithm, a GSS of at least 3.5 is advised for the first-line therapy in a patient carrying a virus with TDR. Abbreviations: ART antiretroviral therapy, 3TC lamivudine, ABC abacavir, ATV atazanavir, ATV/r ritonavir-boosted atazanavir, AZT zidovudine, D4T stavudine, DDI didanosine, DRV/r ritonavir-boosted darunavir, EFV efavirenz, FPV/r ritonavir-boosted fosamprenavir, FTC emtricitabine, IDV indinavir, LPV/r ritonavir-boosted lopinavir, NFV nelfinavir, NVP nevirapine, TDF tenofovir disoproxil fumarate. (DOCX) [file pone.0101738.s002.docx]

**Table S2: Impact of transmitted drug resistance (TDR) on clinical care:** The genotypic susceptibility score (GSS) of each sequence with TDR was calculated for the antiretroviral regimens most frequently prescribed in the year of diagnosis (top three). For instance, the GSS was less than 3 for each of the most frequently prescribed regimens in the only sequence with TDR sampled in 1998. According to the Rega algorithm, a GSS of at least 3.5 is advised for the first-line therapy in a patient carrying a virus with TDR. Abbreviations: ART antiretroviral therapy, 3TC lamivudine, ABC abacavir, ATV atazanavir, ATV/r ritonavir-boosted atazanavir, AZT zidovudine, D4T stavudine, DDI didanosine, DRV/r ritonavir-boosted darunavir, EFV efavirenz, FPV/r ritonavir-boosted fosamprenavir, FTC emtricitabine, IDV indinavir, LPV/r ritonavir-boosted lopinavir, NFV nelfinavir, NVP nevirapine, TDF tenofovir disoproxil fumarate.

| **Number of sequences with TDR per year (%)** | | | | | | | | | | | | | | | |
| --- | --- | --- | --- | --- | --- | --- | --- | --- | --- | --- | --- | --- | --- | --- | --- |
| **YEAR** | **1998** | | | **1999** | | | **2000** | | | **2001** | | | **2002** | | |
| **GSS/ ART** | D4T DDI NFV | D4T 3TC NFV | AZT 3TC NFV | AZT 3TC NFV | D4T DDI NFV | D4T 3TC NFV | AZT 3TC NFV | D4T 3TC ATV | D4T 3TC NFV | AZT 3TC NFV | AZT DDI NFV | AZT 3TC NVP | AZT 3TC LPV/r | AZT 3TC NFV | AZT 3TC EFV |
| ≥ 3.5 |  |  |  |  |  |  |  |  |  |  |  |  | 2 (66.7) |  | 1 (33.3) |
| 3 |  |  |  |  |  |  | 0 (0) | 0 (0) | 0 (0) |  |  |  |  | 2 (66.7) | 2 (66.7) |
| <3 | 1 (100) | 1 (100) | 1 (100) | 3 (100) | 3 (100) | 3 (100) |  |  |  | 3 (100) | 3 (100) | 3 (100) | 1 (33.3) | 1 (33.3) |  |
| **YEAR** | **2003** | | | **2004** | | | **2005** | | | **2006** | | | **2007** | | |
| **GSS/ ART** | AZT 3TC LPV/r | AZT 3TC EFV | AZT 3TC NFV | AZT 3TC EFV | ABC 3TC FPV/r | TDF 3TC LPV/r | AZT 3TC LPV/r | AZT 3TC EFV | TDF 3TC EFV | TDF 3TC EFV | ABC 3TC EFV | TDF FTC LPV/r | TDF FTC LPV/r | TDF 3TC EFV | ABC 3TC EFV |
| ≥ 3.5 |  |  |  |  | 5 (71.4) | 5 (71.4) | 2 (33.3) |  |  |  |  | 3 (75.0) | 11 (84.6) |  |  |
| 3 | 2 (100) |  |  | 3 (42.9) |  |  | 2 (33.3) | 2 (33.3) | 6 (100) | 4 (100) | 4 (100) |  |  | 10(76.9) | 10 (76.9) |
| <3 |  | 2 (100) | 2 (100) | 4 (57.1) | 2 (28.6) | 2 (28.6) | 2 (33.3) | 4 (66.7) |  |  |  | 1 (15.0) | 2 (15.4) | 3 (23.1) | 3 (23.1) |
| **YEAR** | **2008** | | | **2009** | | | **2010** | | | **2011** | | | **2012** | | |
| **GSS/ ART** | TDF 3TC EFV | TDF 3TC LPV/r | AZT 3TC LPV/r | TDF FTC EFV | TDF FTC LPV/r | TDF FTC ATV/r | TDF FTC EFV | TDF FTC ATV/r | TDF FTC DRV/r | TDF FTC EFV | TDF FTC ATV/r | TDF FTC DRV/r | TDF FTC ATV/r | TDF FTC EFV | TDF FTC DRV/r |
| ≥ 3.5 |  | 6 (85.7) | 6 (85.7) |  | 7 (77.8) | 7 (77.8) |  | 6 (85.7) | 7 (100) |  | 6 (85.7) | 7 (100) | 3 (100) |  | 3 (100) |
| 3 | 1 (14.3) |  |  | 3 (33.3) | 1 (11.1) | 1 (11.1) | 5 (71.4) |  |  | 6 (85.7) |  |  |  | 3 (100) |  |
| <3 | 6 (85.7) | 1 (14.3) | 1 (14.3) | 6 (66.7) | 1 (11.1) | 1 (11.1) | 2 (28.6) | 1 (14.3) |  | 1 (14.3) | 1 (14.3) |  |  |  |  |
